# Mesenchymal stem cell transplantation in burn wound healing: uncovering the mechanisms of local regeneration and tissue repair

**DOI:** 10.1007/s00418-023-02244-y

**Published:** 2023-10-17

**Authors:** Mohamed E. El-Sayed, Ahmed Atwa, Ahmed R. Sofy, Yasser A. Helmy, Khaled Amer, Mohamed G. Seadawy, Sayed Bakry

**Affiliations:** 1 Zoology Department, Faculty of Science (Boys), Al-Azhar University, Cairo, 11884 Egypt; 2 Botany and Microbiology Department, Faculty of Science, Al-Azhar University, Nasr City, Cairo, 11884 Egypt; 3 Department of Plastic & Reconstructive Surgery, Faculty of Medicine, Al-Azhar University, Cairo, Egypt; 4 Egypt Center for Research and Regenerative Medicine, ECRRM, 3A Ramses Extension St., Cairo, 11759 Egypt; 5 Biological Prevention Department, Ministry of Defense, Cairo, 11766 Egypt; 6 Center for Genetic Engineering- Al-Azhar University, Nasr City, Cairo, 11884 Egypt

**Keywords:** Mesenchymal stem cells (MSCs), Thermal injuries, Inflammatory cytokines, Histopathology

## Abstract

Burn injuries pose a significant healthcare burden worldwide, often leading to long-term disabilities and reduced quality of life. To explore the impacts of the transplantation of mesenchymal stem cells (MSCs) on the healing of burns and the levels of serum cytokines, 60 fully grown Sprague–Dawley rats were randomly divided into three groups (*n* = 20 each): group I (control), group II (burn induction), and group III (burn induction + bone marrow (BM)-MSC transplantation). Groups II and III were further divided into four subgroups (*n* = 5 each) based on euthanasia duration (7, 14, 21, and 28 days post transplant). The experiment concluded with an anesthesia overdose for rat death. After 7, 14, 21, and 28 days, the rats were assessed by clinical, laboratory, and histopathology investigations. The results revealed significant improvements in burn healing potentiality in the group treated with MSC. Furthermore, cytokine levels were measured, with significant increases in interleukin (IL)-6 and interferon alpha (IFN) observed, while IL-10 and transforming growth factor beta (TGF-β) decreased at 7 days and increased until 28 days post burn. Also, the group that underwent the experiment exhibited increased levels of pro-inflammatory cytokines and the anti-inflammatory cytokine IL-10 when compared to the control group. Histological assessments showed better re-epithelialization, neovascularization, and collagen deposition in the experimental group, suggesting that MSC transplantation in burn wounds may promote burn healing by modulating the immune response and promoting tissue regeneration.

## Introduction

The problem of burn wounds caused by intentional injuries is significant, and it remains challenging to find effective treatments. Burn wounds can vary in severity and are caused by several sources such as heat, chemicals, electricity, radiation, and friction (Mirshekar et al. 2023). The World Health Organization (WHO) reported that 265,000 people worldwide die from burns every year, and non-fatal burns are a major cause of disease (Nielson et al. 2017; WHO 2017; Mehta et al. 2022).

Many pharmacological and non-pharmacological strategies have been tried and investigated in the treatment of burns with varying degrees of success (Ali and Ali 2022).

Despite significant breakthroughs in treatment procedures for the management of patients with severe burns, such as better resuscitation, improved wound covering, infection control, and inhalation injury management, the consequences of a severe burn are severe (Wolf and Arnoldo 2012; Abdallah et al. 2023).

During the burn injury phases, many cellular and immunological dysfunctions happen. That has led scientists and physicians to develop more and more treatment approaches for better burn patient care and better outcomes with fewer post-treatment disabilities (Schwacha 2003). The pioneering and most recent branch of health and medical sciences dealing with this situation is regenerative medicine (Mason and Dunnill 2008; Atwa et al. 2022).

Stem cells are the premier origin of regenerative medicine techniques used to regenerate organs and resolve tissue abnormalities caused by age-related effects and congenital disabilities (Abdel-Gawad et al. 2021). Regenerative medicine, utilizing stem cell therapy, is employed in burn therapy to hasten the process of re-establishing functional skin with hair follicles, sweat glands, and skin capillaries. The objective is to accelerate the re-epithelialization and reconstruction of the affected area (Lee et al. 2009).

Most obtainable skin substitutes consist of keratinocytes and fibroblasts and, therefore, cannot induce distinguished structures, including sweat glands and hair. The utilization of recently discovered cellular varieties like mesenchymal stem cells (MSCs), endothelial cells, and induced pluripotent stem cells (iPSCs) in the development of synthetic skin replacements has displayed encouraging outcomes (Abdul Kareem et al. 2021; Chogan et al. 2023). In addition, the distinctiveness of stem cells and their ability to regenerate themselves and differentiate into various cell types make them capable of regenerating and restoring tissue in distinctive manners (Atwa et al. 2022; Chogan et al. 2023).

Several studies have clarified that MSC therapy rapidly effects and accelerates re-epithelialization in radiation and thermal burns after removal of eschar (Rodgers and Jadhav, 2018; Smith et al. 2010), inducement of angiogenesis through hepatocyte growth factor (HGF) and vascular endothelial growth factor (VEGF)-A production (Nie et al. 2011), expression of anti-inflammatory molecules, and immunomodulation (Regmi et al. 2019). The therapeutic effect of MSCs in reperfusion injuries/ischemia can also be demonstrated by the production of growth factors (Xing et al. 2014) and the suppression of inflammatory cell induction, recruitment, and efficiency (Li et al. 2018). The important information regarding the beneficial impact of MSCs in addressing serious injuries that involve complicated pathophysiological characteristics justifies using MSC-focused treatments and therapies for frostbite (Durymanov et al. 2020).

Although stem cells are known for their multipotency, recent studies have emphasized that the potency of MSCs is primarily determined by the secretion of various factors, including fibroblast growth factor (FGF), insulin-like growth factor (IGF), platelet-derived growth factor (PDGF), transforming growth factor (TGF) beta, interleukin (IL)-10, IL-4, and IL-6. Although these factors play crucial roles in cell recruitment, immune regulation, wound healing, and angiogenesis, rather than in cell lineage (Maacha et al. 2020; Miceli et al. 2021; Peta et al. 2021), there are no up-to-date studies that target the effect of MSCs on wound healing regarding clinical, laboratory, and histopathology findings. Therefore, our study aimed to shed light on and discuss the effect of locally transplanted MSCs on wound healing of deep second-degree burns in rats regarding clinical, laboratory, and histopathology investigations. A skin island burn wound model was developed in rats to assess wound healing, skin revival, and regeneration after local and topical injection of MSCs.

## Material and methods

### Animal care

The research plan received approval from the Fayoum University Institutional Animal Care and Use Committee (FU-IACUC) under Code No. AEC 2303, in accordance with the Global Strategies for the Ethical Treatment and Utilization of Laboratory Animals.

Male Sprague–Dawley rats aged 6 weeks and weighing between 220 and 280 g were acquired from the laboratory of the Animal House, Faculty of Science at Al-Azhar University in Cairo, Egypt. The inhabitants were housed in separate compartments that are divided, and they were provided with consistent resources such as water, food, temperature, light, and humidity. The cycle of light and darkness was maintained at 12 h each. The experimental animals were tagged and homed in cages cleaned daily. A standard rodent pellet regimen manufactured by the Egyptian Company for Oil and Soap and several vegetables were used to feed the animals. In addition, during the periods of the experiments, water was supplied ad libitum. After an acclimatization period of 1 week, the rats were used for the experiments.

### Animal model of severe burn and experimental design

Sixty Sprague–Dawley rats that were fully grown were randomly divided into three groups, each with 20 rats: group I control group (sham), group II underwent burn induction, and group III underwent burn induction followed by local transplantation of bone marrow-derived (BM)-MSCs specifically in the right burn area.

Groups II and III were equally divided into four subgroups (five rats per subgroup) depending on the duration of euthanasia at 7, 14, 21, and 28 days after cell transplantation. At the end of the experiment, all rats were killed with an anesthetic overdose.

A rat skin burn model was designed, and intraperitoneal injection (i.p.) of a mixture of xylazine/ketamine (XK) (5 mg/kg i.p.; 50 mg/kg i.p.) was used to anesthetize the rats (Ritschl et al. 2015). On the backs of these rats, dorsal hair was completely removed. First, hair was cut with clippers, and then a chamomile-enriched hair removal cream was applied for sensitive skin. Next, using an aluminum punch, two circular burns with a diameter of 20 mm were made on each rat’s right and left dorsal side. In this experiment, the aluminum stamp underwent boiling in water at a temperature of 100 °C for a duration of 30 s. After this, the stamp was placed on each side for 10 s without any pressure applied. Each rat in the study had two circular burns, each with a diameter of 20 mm and extending through the full thickness of the skin. To prevent shock, each rat received an injection of balanced salt solution, with a dose of 40 mg/kg of body weight. The burn wounds on the back of the rats were then treated with a solution containing 1% tincture of iodine to promote healing and prevent infection. The wounds were kept dry throughout the study. The rats in the control group underwent identical procedures, with the exception that they were submerged in water at a temperature of 37 °C for 10 s.

### Assessment of wound closure rate

Weekly measurements of wound closure were conducted using a digital caliper to determine the extent of occlusion of the length of the burn diameter on both sides of each rat. The measurement was recorded as a numeric value. In addition, digital photographs of the wound were taken from 10 cm distance using a 12-MP digital camera with a 3-inch LCD, keeping the lens parallel to the burn wound. The EOS 4000D DSLR camera used was manufactured by Canon Inc. in Tokyo, Japan.

### Isolation and culturing of MSCs from rat bone marrow

BM-MSCs were extracted from the bone marrow of male Sprague–Dawley rats aged 4 weeks, using the methodology previously outlined (Nakano et al. 2016; Chaudhary and Rath 2017; Ahmed et al. 2021). To summarize, the rats were anesthetized, and their femurs and tibiae were collected and cleaned to remove muscle and connective tissue. The bone marrow was then rinsed and grown in a solution of low-glucose DMEM with 10% fetal bovine serum (FBS, Invitrogen Australia Pty Ltd., Mount Waverley, Victoria, Australia) and 1% penicillin/streptomycin, after the epiphysis was removed. The bone marrow was kept in a humidified, 5% CO_2_ environment at 37 °C. The medium was changed twice a week until the cells grew to almost 80% confluence. The MSCs used in all experiments were from the third and fourth passages.

### Immunophenotyping of isolated bone marrow cells

To commence the process, MSCs in their third passage underwent a sequence of steps, encompassing rinsing, refining, and exposure to solutions containing antibodies. These antibodies were specifically designed to target distinct cell markers: CD90, CD73, CD105, and CD34. The solutions were formulated using a 0.5% FBS base. Antibodies for CD34, CD73, CD90, and CD105 intended for flow cytometry were obtained from Becton–Dickinson Biosciences (BD Stemflow, Piscataway, NJ). Subsequently, the cells were treated with the antibody solutions in the absence of light at 4 °C for a duration of 30 min.

The fluorescence of the cells was immediately analyzed using a FACS Calibur (Cyto-FLEX) flow cytometer from Becton–Dickinson, San Jose, CA, USA, and the data were processed using Cell Quest software (Becton–Dickinson). Isotype-identical antibodies were employed as control samples as described elsewhere (Nacer Khodja et al. 2013; Boxall and Jones 2015; Liu et al. 2020).

### Localized injection of BM-MSCs

Each right burn circle for the animal within group III received (1 × 10^6^) cells from BM-MSCs in 500 µL PBS transplanted at four injection sites localized around the wound, while the left burn circle was left without any treatment.

### Histopathological assessment

The Heidenhain’s Susa solution was utilized to preserve skin samples from normal and wounded rats. The process involved two steps: fixation and dehydration. Fixation included submerging tissues in 10% buffered formalin for 48 h, followed by a 30-min distilled water rinse. Dehydration used a sequence of alcohol solutions (70%, 90%, and 100%). After that, samples were cleared with xylene, impregnated with paraffin wax, and sectioned (4–5 µm) for staining as described by (Suvarna et al. 2018) to identify various tissue abnormalities.

### Pro- and anti-inflammatory cytokine analysis in serum blood

Blood samples were collected and analyzed using the enzyme-linked immunosorbent assay (ELISA) technique (Dynatech Microplate Reader model MR 5000) to determine the levels of various markers including interferon (IFN), tumor necrosis factor alpha (TNF-α), TGF, granulocyte–macrophage colony-stimulating factor (GM-CSF), and interleukins IL-6 and IL-10. These levels were measured using Microplate Reader RT-2100C ELISA kits designed for rats that are supplied by Sun Long Biotech Co, Hang Zhou, China.

### Statistical methods

The research employed a completely random design (CRD) and utilized SPSS statistical analysis (version 28.00; IBM Corp, Armonk, NY, USA). Fisher’s test was utilized with a 95% confidence interval for one-way analysis of variance (ANOVA) and *t* test. The analytical data had a quantitative nature, resulting in a parametric distribution of Levene’s test. The heat map displays the examination of Pearson’s correlation and discrimination analysis.

## Results

### In vitro cell study results

The findings from the microscopic and immunophenotyping tests demonstrated that the rat BM-MSCs had similar profiles of surface antigens, morphology, and capacity for multi-differentiation. These characteristics met the minimum standards established by the International Society for Cellular Therapy (ISCT) for MSCs (Dominici et al. 2006).

#### Plastic-adherent fibroblast-like cells

**Fig. 1:**
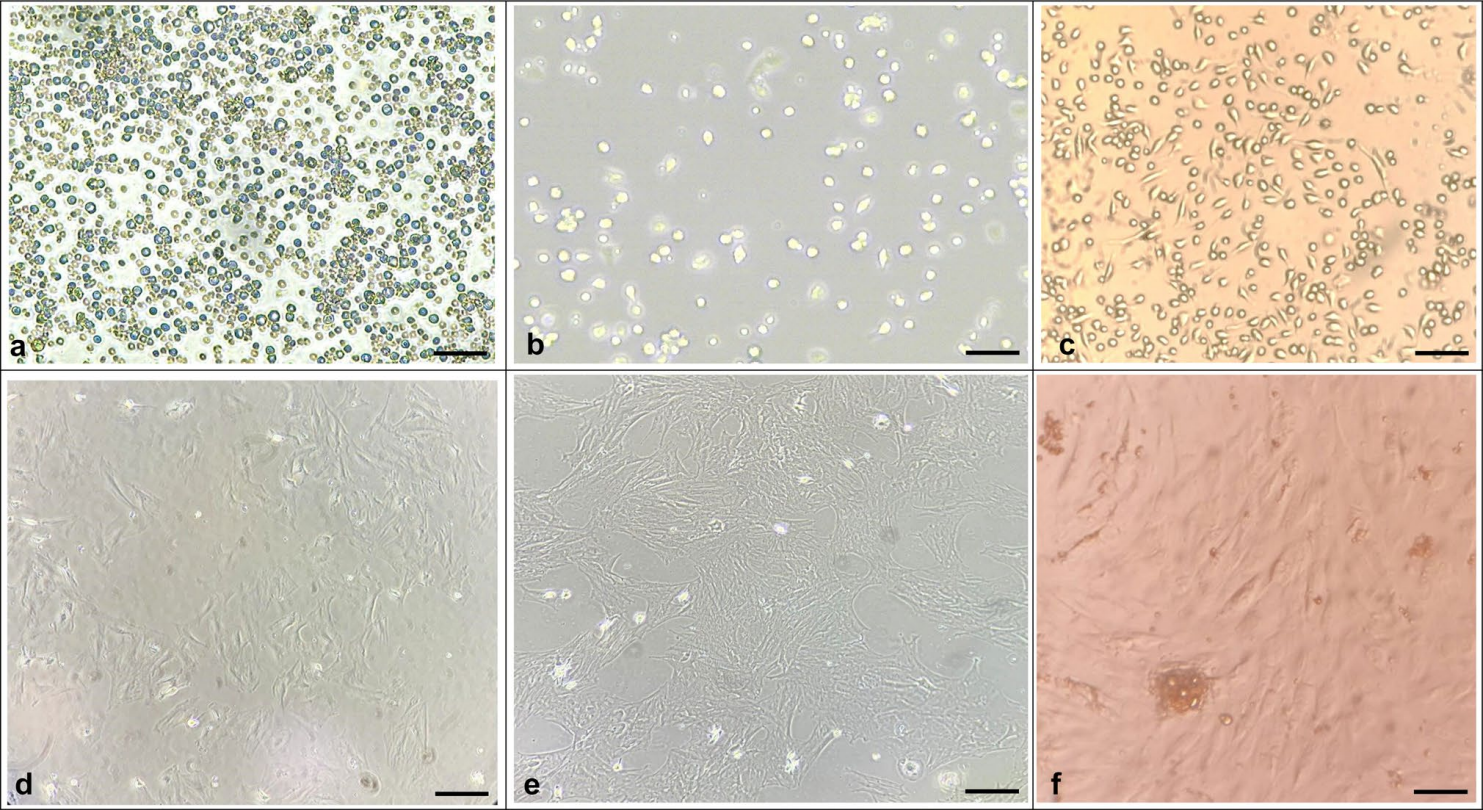
Morphology of mesenchymal stem cell changes with varying passages. The culture and expansion of mesenchymal stem cells (MSCs) derived from rat bone marrow are described in the following images. Image **a** shows the morphology of bone marrow cells (BMCs) under a bright field microscope on day 0, just 10 min postseeding. Image **b** depicts passage 0 (P0) cells, 48 h after initial seeding. Image **c** shows passage 0 (P0) cells 48 h after the first change of the medium. Image **d** displays passage 0 (P0) cells on day 5. Image **e** shows passage 1 (P1) cells on day 11. Image **f** represents passage 2 (P2) cells (scale bar 100 µm)

Following 24 h of being cultured in a standard growth medium at 37 °C, some of the separated cells from the bone marrow showed adherence to the plastic flask’s surface. When the medium was replaced, these cells congregated to form colony-forming units after 5 days of culturing. Over time, the cells’ appearance transformed from round to a fibroblast-like morphology, where they took on a spindle-shaped appearance. Despite this, there were a range of appearances observed, with elongated cells and multipolar projections evident. By the end of 2 weeks in culture, the cells adopted a fingerprint-like orientation and achieved confluency. After cell passage, the cells appeared more homogeneous. In passage 2, they showed a spindle-shaped morphology that could be monitored by light microscopy (Fig. 1).

#### Immunophenotype expression of rat BM-MSCs

**Fig. 2:**
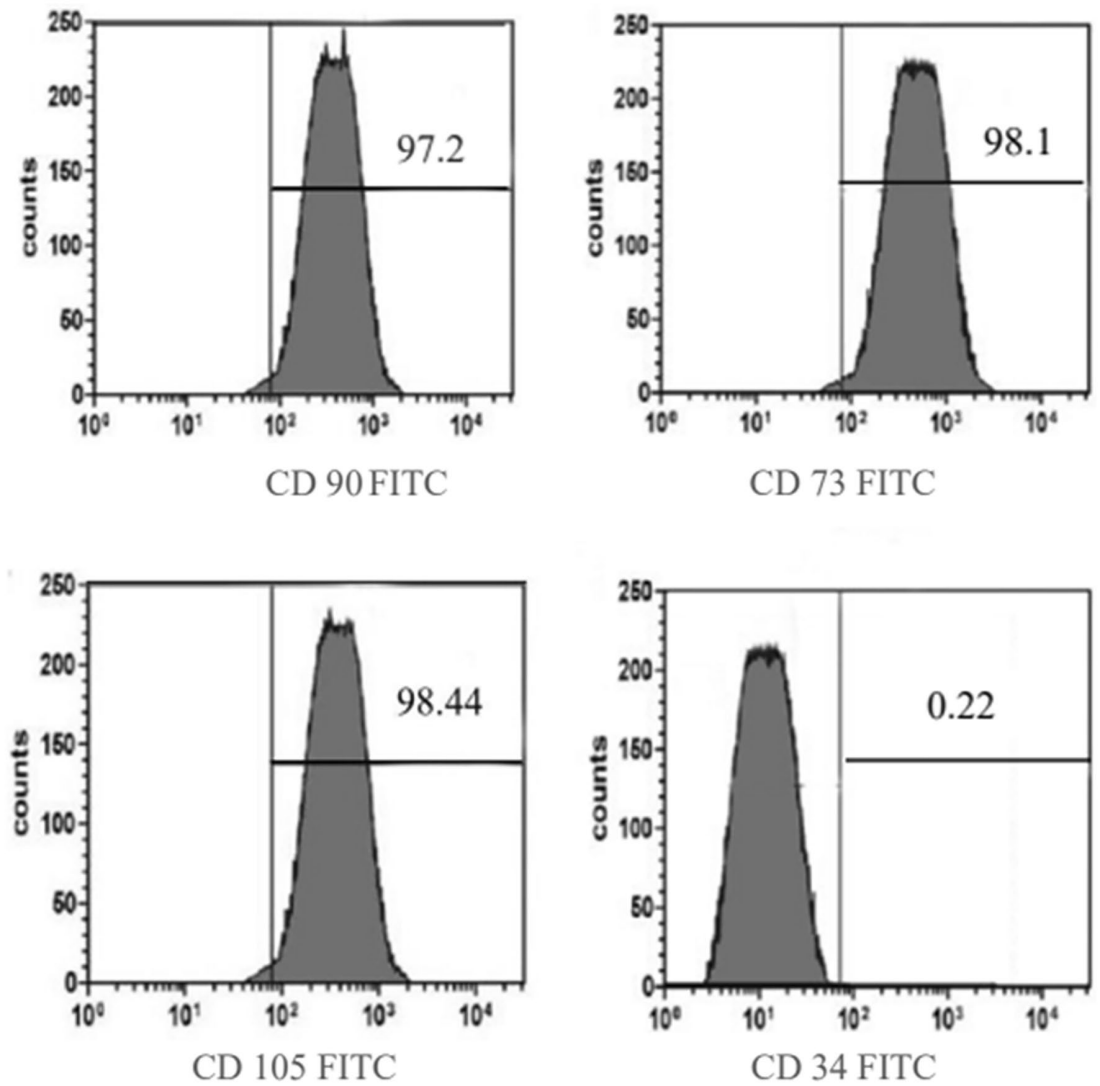
Analysis of the surface of rat BM-MSCs using flow cytometry to determine their immunophenotypic characteristics. The flow cytometry process involved treating cells with antibodies in the dark at 4 °C for 30 min, isotype-identical antibodies served as controls. Results showed high expression of CD73, CD105, and CD90, and negative expression of CD34, indicating non-hematopoietic origin with at least 85% cells showing various marker expressions

The BM-MSCs were cultured and passaged three times. Subsequently, flow cytometry and differentiation assays were conducted to assess their characteristics. The results showed high expression levels of positive surface markers for CD73, CD105, and CD90, while CD34 marker expression was negative. This indicates that the cells were not derived from hematopoietic or leukocyte sources (Fig. 2). The cells were then transplanted into rats for further studies.

### In vivo evaluation of burn wound healing

#### Macroscopic observations and gross evaluation of burn area

**Fig. 3:**
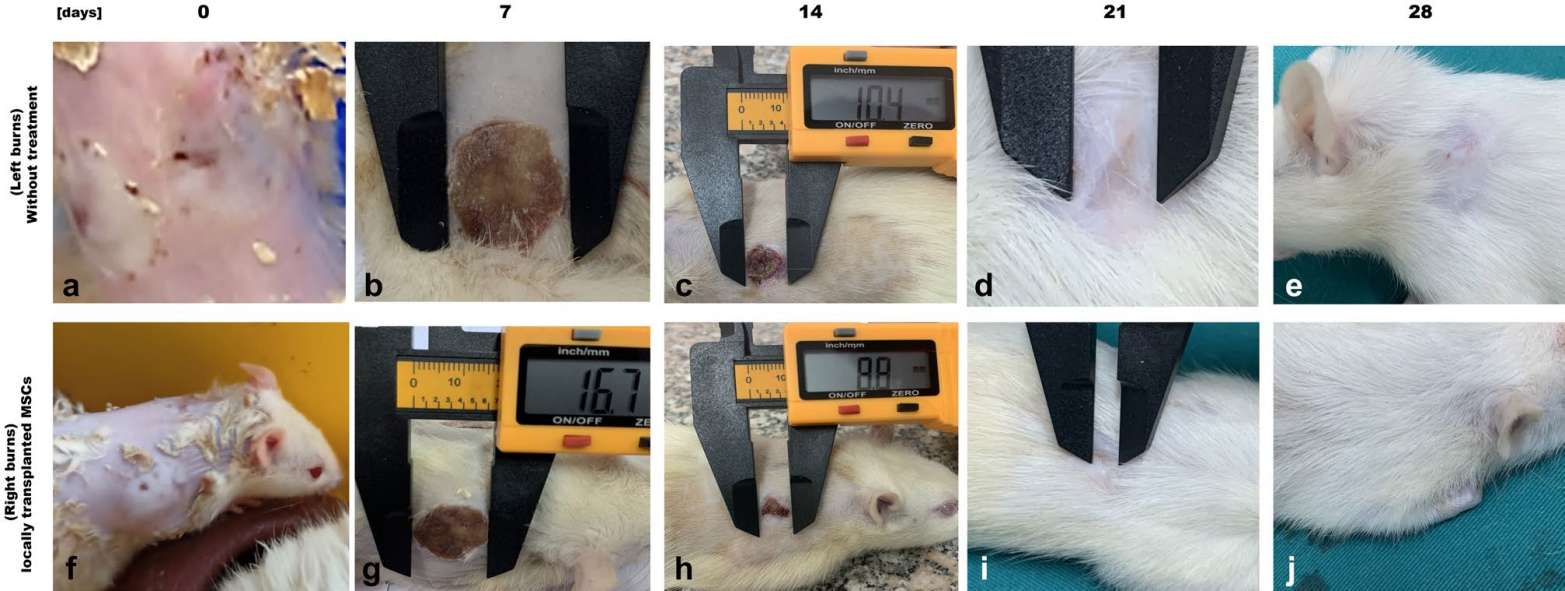
Analysis of burn injuries using a macroscopic method. To analyze burn injuries, the tissue was photographed on days 0, 7, 14, and 28. Images **a**–**e** represent untreated burns induced on the left side, while** f**–**j** depict locally transplanted burned skin on the right side. These images indicated that scar formation occurred by day 7 and a minor detachment was observed by day 14. Furthermore, the burn wound tissues were subjected to transplantation using mesenchymal stem cells (MSCs) and were also examined on days 0, 7, 14, and 28 following the transplantation procedure. On day 7, all the groups that underwent transplantation exhibited wound progression and scab formation. By day 14, a thick scab had formed, covering the entire burn area, with no signs of infection in the MSC-transplanted group. On day 21, complete scab detachment with slight granulation tissue was observed in the MSC-transplanted group, and on day 28, complete scab detachment, re-epithelialization, and the emergence of hair growth were observed

After the animals recovered from anesthesia, they appeared comfortable. After pyrolysis, grossly pale circular scars were observed in both treated and untreated animals. Blisters appeared at 2 h, which became pale at 6 h, and ruptured blisters developed at 12 h, significantly increasing to 24 h in animals treated with BM-MSCs. On the first day, some of the wounds were damp and had some leakage, but by the third day, most of them were beginning to dry up. Initially, there was some slight oozing, but this gradually decreased and by day 3, there was no oozing observed in any of the groups. After 7 days, a firm and reddish scab formed over the wound, and it became smaller in size. This scab remained attached to the wound for up to 14 days in only one burned animal. However, in the animals that received treatment, the wound was sensitive, and the skin appeared to be returning to a nearly normal state. The initial wound area was almost 20 mm in diameter at day 0 after wound generation (Fig. 3). The findings indicate that wound healing is enhanced by MSCs, as the percentage of wound closure in the group treated with MSCs is consistently higher compared to the untreated burn group.

A *t* test analysis was used to determine if the observed variations between groups are statistically meaningful. The *P* value is an indicator of the likelihood that the detected differences between groups are a result of random chance. In most cases, a significance level of 0.05 or lower for a *P* value is deemed significant from a statistical standpoint. This implies that the probability of the observed difference being a result of random chance is less than 5%. In this case, for all the time points, the *P* values were less than 0.05, which suggests that the observed variations between the groups are statistically significant (Table 1). In addition, the *t* test offers insights into the extent of variation between the groups. The *t* value signifies the number of standard deviations that separate the means of the two groups. A higher *t* value reflects a greater disparity between the groups. Upon examination of the* t* values, it is evident that the disparity between the untreated burn group and the MSC-treated group is more prominent during later time points (21 and 28 days) than at earlier time points (7 and 14 days). Overall, the results suggest that locally transplanted MSCs positively affect burn wound healing, and the effect is more prominent at later stages of wound healing.

**Table 1:** Percentage of the burn wound’s contracture (closure) rate for the burn group and the locally transplanted burns in the MSCs group

Time points	Groups		*t* test	*P* value	
	Burn	MSCs			
7 days burn (*n* = 5)	18.36 ± 0.4	16.84 ± 0.43	2.608	0.018	S
14 days burn (*n* = 5)	10.96 ± 0.36	8.83 ± 0.26	4.752	< 0.001	HS
21 days burn (*n* = 5)	9.2 ± 0.3	5.19 ± 0.21	10.941	< 0.001	HS
28 days burn (*n* = 5)	6.79 ± 0.37	0.13 ± 0.002	18.353	< 0.001	HS

A Comparative Analysis of Burn Wound Contracture Rates Over Four Time Intervals (7, 14, 21, and 28 Days) Between the Burn Group and the Stem Cells Group

Table 1 presents a comparative analysis of burn wound contracture rates observed over four distinct time intervals (7, 14, 21, and 28 days) between two distinct groups, namely the burn group (*n* = 20) and the stem cells group (*n* = 20). Each of these groups was further subdivided into four subgroups, each consisting of 5 individuals at each time point. The statistical analysis employed for this investigation was a* t* test, assessing the differences in contracture rates between the two groups. The results demonstrate statistically significant variations between these groups, as indicated by *p* values (S denotes significance at *p* value < 0.05, HS denotes high significance at *p* value < 0.001). The findings provide valuable insights into the variations in burn duration across the groups and underscore the importance of further investigation and interpretation of these differences

#### Microscopic (histopathological) investigations

##### Histology of control and burn groups

**Fig. 4:**
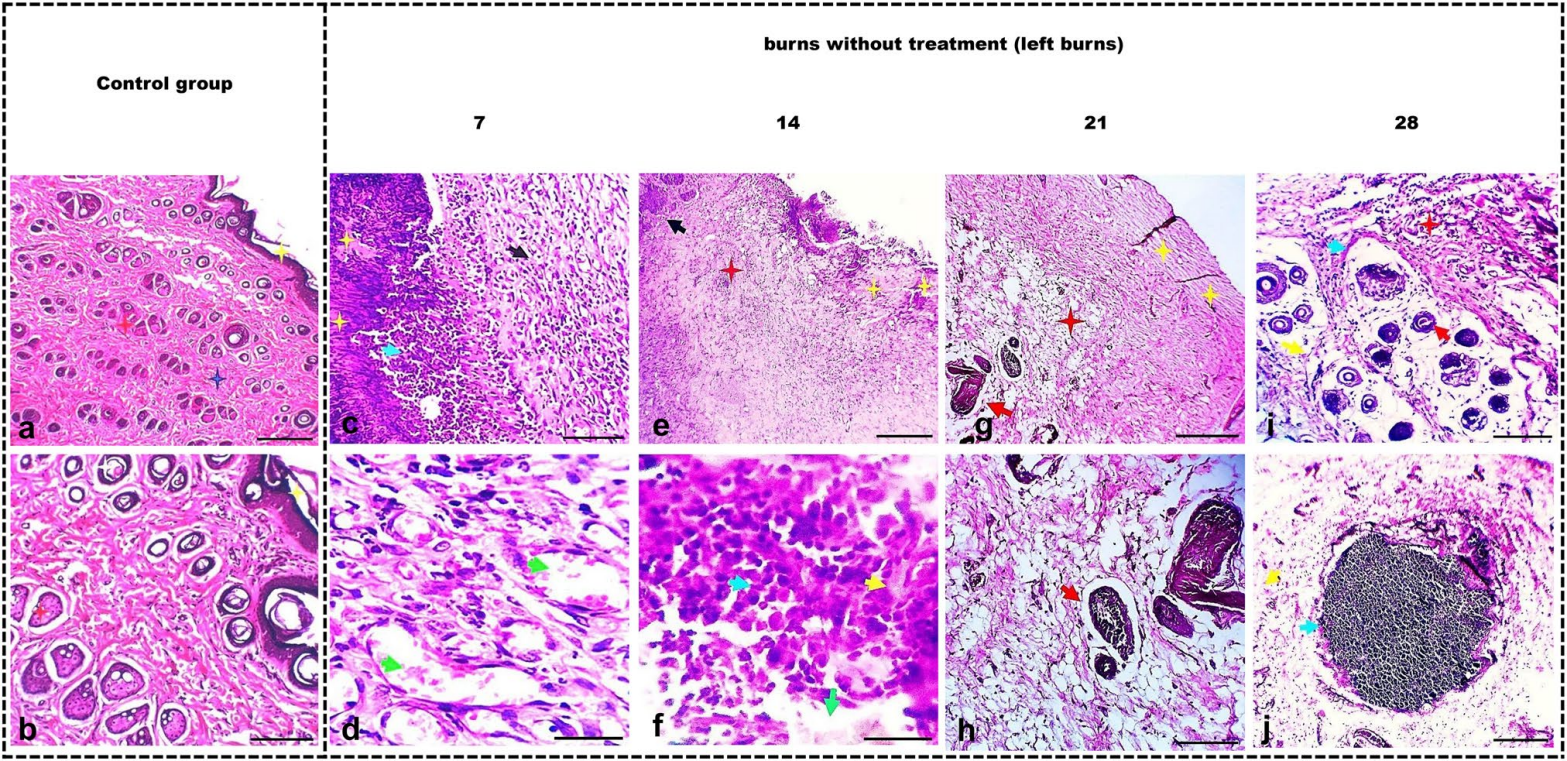
Photomicrograph of the skin from both control rats and untreated burned skin in groups II and III. The skin of rats in the control group (**a**, **b**) exhibits typical epidermal features, including a noticeable basal layer (orange star), an indistinct spinous layer, a subtle granular layer (green star), and varying thickness of the outermost layer (yellow star). The dermal layer contains tightly packed collagen fibers (dark blue star), and there are visible groups of small and large hairs along with sebaceous glands (white and red stars). In the burn group after 7 days (**c**, **d**) ulcerative lesions present with destruction of the epidermis, intense tissue necrosis, and fragmentation (yellow star and arrowhead). The dermis shows failed tissue remodeling with mixed inflammation-infiltrated granulation tissue formation (black arrowhead). Collagen deposition partially failed, and collagen fibers appear haphazardly arranged (black arrow). Dermal arterioles and venules exhibit arteriolitis and phlebitis (green arrowhead). Skin appendages are missing, and focal granulomatous reactions with some giant cells are present (red arrowhead). After 14 days (**e**, **f**) epidermal layers are destroyed, replaced by necrotic debris, fluid, and inflammation (yellow stars and arrowhead). Hypodermal tissue near the ulcer shows ischemic changes, lymphocyte infiltration (blue arrowhead), hemorrhagic spots (green arrowhead), and less collagen deposition (red stars). Skin appendages are damaged by inflammation (red arrowhead). Dermal tissue at wound margins is mildly hypertrophic (black arrowhead). After 21 days (**g**, **h**) ulcer with fluid, cellular exudate of neutrophils and debris are observed as well as mild inflammation and collagen disorganization, edema, mixed inflammation, and destruction of hair follicles and glands in the dermis. Furthermore, there is a mild to moderate hyperemia in the blood vessels. At 28 days (**i**, **j**) ulcerative lesions with minimal exudate, scanty inflammatory cells (mostly round), and moderate hypodermal inflammation with edema, cellular infiltration, and collagen fiber disintegration are visible. Scale bars 100 μm (**a**, **c**, **e**, **g**, **i**), 25 μm (**b**, **d**, **f**, **h**, **j**)

The skin burn sections were subjected to H&E staining, which revealed that in rats of the control group, the epidermis appeared normal with discernible layers and an outermost layer of varying thickness. The dermis exhibited tightly packed collagen bundles, sebaceous glands, and hair follicles. Additionally, the skin had nerves and vessels located at the dermis base along with arrector pili muscles (Fig. 4).

In the burn group, after 7 days, ulcerative lesions present with the destruction of the epidermis, intense tissue necrosis, and fragmentation (Fig. 4). The dermis shows failed tissue remodeling with mixed inflammation-infiltrated granulation tissue formation. Collagen deposition partially fails, and collagen fibers appear haphazardly arranged. Dermal arterioles and venules exhibit arterioles and phlebitis. Skin appendages are missing, and focal granulomatous reactions with some giant cells are present. After 14 days, epidermal layers are destroyed, replaced by necrotic debris, fluid, and inflammation (Fig. 4). Hypodermal tissue near the ulcer had ischemic changes, lymphocyte infiltration, hemorrhagic spots, and less collagen deposition. Skin appendages were damaged by inflammation. Dermal tissue at wound margins was mildly hypertrophic.

After 21 days, the ulcer represents with fluid, cellular exudate of neutrophils, and debris. Mild inflammation and collagen disorganization was observed, as well as edema, mixed inflammation, and destruction of hair follicles and glands in the dermis. A mild to moderate hyperemia in blood vessels was visible (Fig. 4).

At 28 days, ulcerative lesion with minimal exudate, scanty inflammatory cells (mostly round), and moderate hypodermal inflammation with edema, cellular infiltration, collagen fiber disintegration, skin appendage degeneration, and nodular aggregate were observed (Fig. 4).

##### Transplantation of mesenchymal cells promotes development of blood vessels and granulation tissue, while reducing presence of inflammatory cells within wound sites

**Fig. 5:**
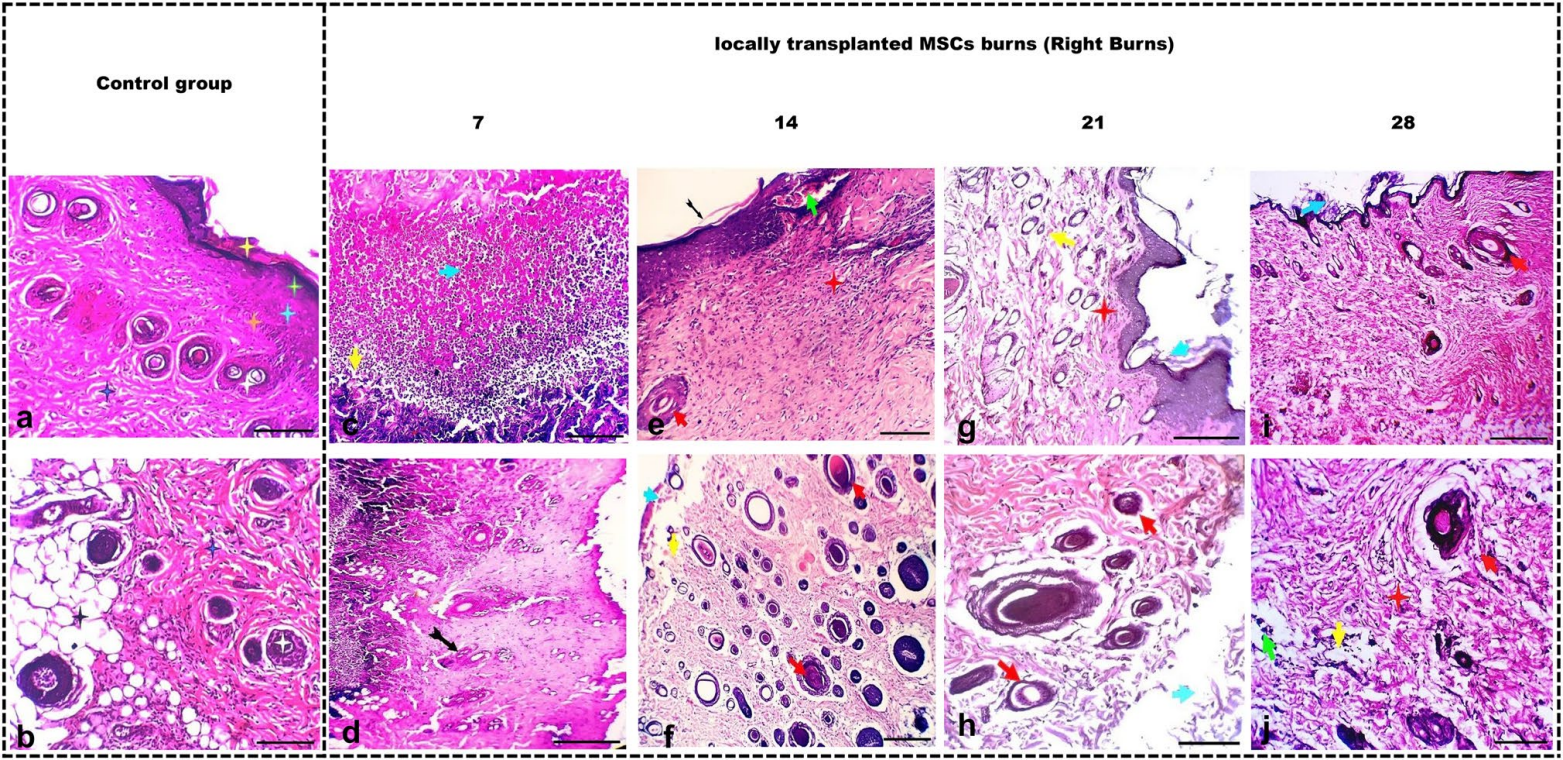
Photomicrographs for the control skin in comparison with MSCs transplanted burns. In controls **a**, **b**, the typical epidermal layers are observed, with a distinct stratum basal (orange stars) and granulosum (green stars), varying stratum corneum thickness (yellow stars), and dense collagen fibers in the dermis (dark blue star), alongside vellus and guard hairs (white star) with sebaceous glands (red stars). **c**, **d** Show a rat’s skin 7 days after stem cell therapy for an induced wound. The images reveal massive destruction and necrosis of the dermal tissue with the presence of tissue necrotic debris and dead inflammatory cells (light blue arrowheads). The hypodermal tissue is thin and destructed (yellow arrowhead), while the dermal stroma is edematous and infiltrated with many polymorph leukocytes. Skin appendages are partially or completely damaged, while the subcutaneous tissue shows minimal tissue damage (black arrow). At 14 days, **e**, **f** a partially healed ulcer with hyperkeratosis and healthy tissue underneath is observed (light blue arrowhead). Some hair follicles show degeneration and cystic changes (blue star). Remodeled granulation tissue is replacing the previously damaged tissue (between red star and green arrowhead), but mild hyperemia and inflammatory cells are present (green arrowhead). The surrounding skin appears healthy and nearly fully healed after 21 days (black arrow). **g**, **h** The skin has regenerated and re-keratinized, with healthy tissue and normal hair follicle growth (light blue arrowheads). Some hair follicles have degenerated and show cystic changes and perifollicular edema (blue stars and yellow arrowheads). Immature granulation tissue is replacing the previously damaged tissue, with mild hyperemia and mild dilatation of surrounding blood vessels (green arrowhead). The skin is completely healed 28 days (**i**, **j**) after stem cell therapy. The wound had an ulcerative lesion, but it now shows epidermal epithelial regeneration and re-keratinization (light blue arrowhead). The hypodermal and subcutaneous tissue underneath appears to be healthy, and there is normal growth of hair follicles and sebaceous glands (red arrowheads and stars). The subcutaneous fibrous tissue has undergone remodeling, and there are reparative hyperemic and edematous changes visible (green and yellow arrowheads). Scale bars 100 μm (**a**, **c**, **e**, **g**, **i**), 25 μm (**b**, **d**, **f**, **h**, **j**)

A severe ulcerative lesion with extensive tissue destruction and necrosis was observed 7 days after stem cell therapy. Still, the underlying subcutaneous tissue appeared minimally damaged (Fig. 5). At 14 days, a partially healed ulcer with hyperkeratosis and healthy tissue underneath was observed. Some hair follicles showed degeneration and cystic changes. Remodeled granulation tissue replaced the previously damaged tissue, but mild hyperemia and inflammatory cells were present. The surrounding skin appeared healthy and nearly fully healed after 21 days. The skin had regenerated and re-keratinized, with healthy tissue and normal hair follicle growth. Some hair follicles had degenerated and showed cystic changes and perifollicular edema. Immature granulation tissue replaced the previously damaged tissue with mild hyperemia and mild dilatation of surrounding blood vessels. They completely healed 28 days after stem cell therapy. The wound had an ulcerative lesion but now showed epidermal epithelial regeneration and re-keratinization. The underlying hypodermal and subcutaneous tissue appeared to be in good condition, with normal growth of hair follicles and sebaceous glands. However, the subcutaneous fibrous tissue had undergone remodeling, and there were noticeable changes in reparative hyperemia and edema.

#### Changes in levels of pro-inflammatory and anti-inflammatory cytokines after transplantation of MSCs into areas affected by burns

All the animals who suffered from burn injuries were able to survive. Furthermore, we analyzed the levels of cytokines in the blood serum of the MSCs, burn, and control groups at 7, 14, 21, and 28 days after the transplantation procedure using an ELISA. The levels of IL-6, IL-10, TGFβ, GM-CSF, TNFα, and INFα in the burn group were compared with those of the MSCs group, burn group only, and control group, as shown in Table 2.

**Table 2:** Table 2 provides a Comparative Analysis of Anti/Pro-inflammatory Cytokine Profiles in the Control Group

Control group cytokine levels:	IFN	TGF-β	GM-CSF	TNF-α	IL-6	IL-10
	16.20 ± 1.31c	468.0 ± 26.1c	12.73 ± 0.81 b	108.0 ± 2.64b	42.33 ± 2.08c	96.00 ± 1.00d

The Control Group, comprising a total of 20 subjects (*n* = 20), underwent evaluation to determine the levels of various cytokines, namely IFN-α, TGF-β, GM-CSF, TNF-α, IL-6, and IL-10. The data in this table is presented as means (± standard error) for each cytokine

Statistical analysis using Fisher’s test at *p* < 0.05 demonstrated significant variations in means (± standard error) across different cytokines, as indicated using distinct letters (a–d) within the same row. This signifies that the levels of these cytokines in the Control Group differ significantly from one another, warranting further investigation and analysis

Note: The use of letters (a–d) on the same row typically indicates statistically significant differences between the means, with different letters signifying different groups or levels of significance

Considerable elevations were noticed in GM-CSF, TNF-α, IL-6, and IFN-α at 7 days following burn injury, but these continued to decline until 28 days. A significant increase was also observed for IL-6 and IFN-α (*p* < 0.001) (Table 3).

**Table 3:** A comprehensive comparative analysis of the levels of six specific cytokines, namely IFN-α, TGF-β, GM-CSF, TNF-α, IL-6, and IL-10, within the context of a controlled study conducted on individuals belonging to a burn group

Time points	IFN-α	TGF-β	GM-CSF	TNF-α	IL-6	IL-10
7 days (*n* = 5)	23.2 ± 0.72a	374.67 ± 2.52e	17 ± 2a	313 ± 6.24a	75.67 ± 4.93b	185 ± 2b
14 days (*n* = 5)	12.83 ± 1.04d	410.33 ± 10.02d	10.17 ± 1.04c	93 ± 2.65c	90 ± 3a	135 ± 5c
21 days (*n* = 5)	17.27 ± 1.22bc	531 ± 10.54b	11.4 ± 1.51bc	94 ± 3.61c	42 ± 2c	91 ± 2.65d
28 days (n = 5)	19.67 ± 2.08b	565.33 ± 5.03a	11.4 ± 0.46bc	84.77 ± 1.08d	92.67 ± 3.06a	244.67 ± 5.03a
*F* ratio	24.65	103.00	12.84	2139.46	182.69	1011.19
*P* value	< 0.001 HS	< 0.001 HS	< 0.001 HS	< 0.001 HS	< 0.001 HS	< 0.001 HS

The data in this table were acquired from a total sample size of 20 participants within the burn group. To enhance the granularity of the analysis, each of the participants was further stratified into four subgroups, comprising 5 individuals each, at four distinct time points: 7 days, 14 days, 21 days, and 28 days post injury

HS indicates high significance at a *p* value of less than 0.001. Fisher’s test at *p* < 0.05 was applied to determine significant differences in means (± SE), which are represented by distinct letters (a–e) within the same row, denoting statistically significant variations between groups

**Fig. 6:**
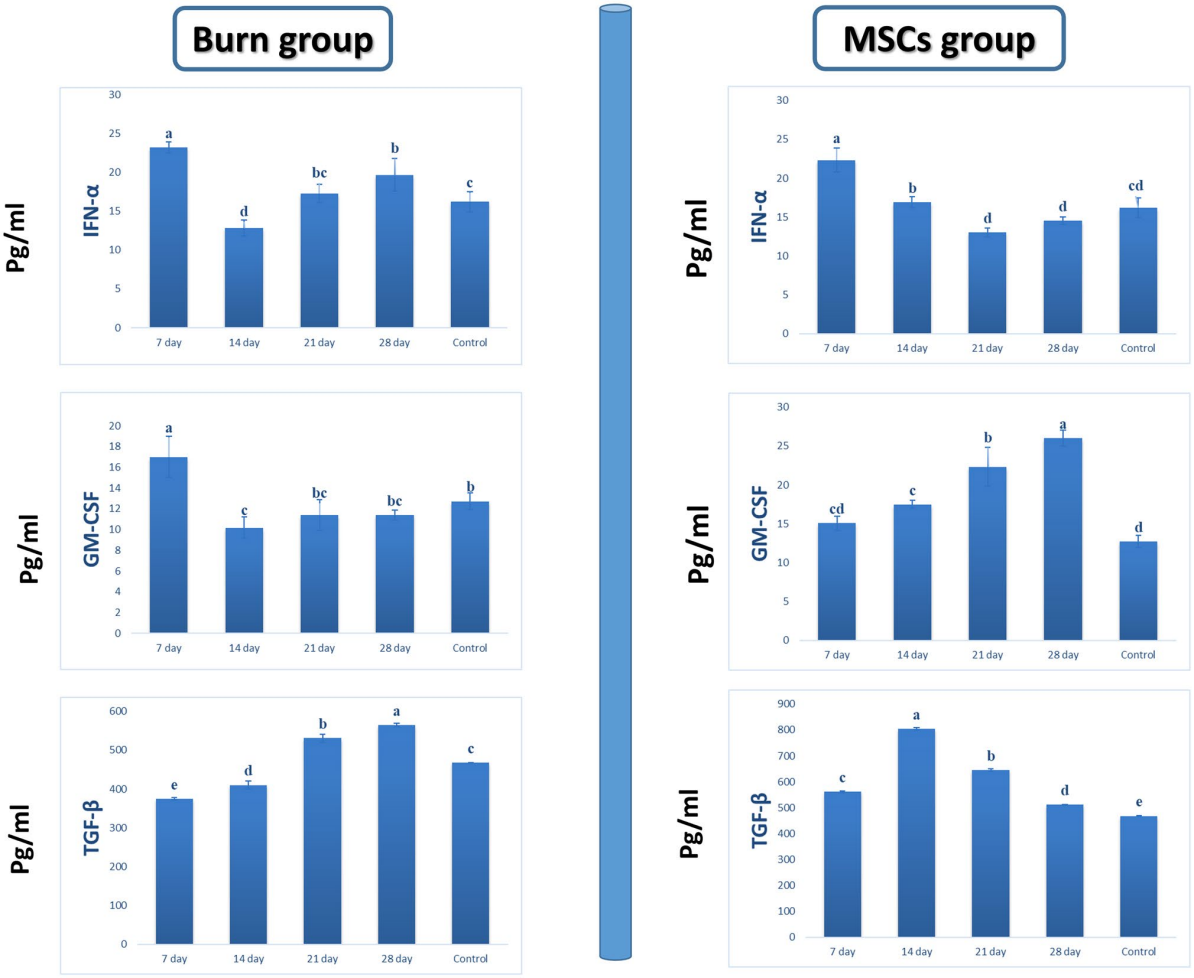
Analysis of growth and differentiation factors in a rat model of burns. Cytokine levels in the blood serum were measured in three rat groups: those with burns but no treatment, those treated with MSCs, and control rats. An ELISA was used to monitor the changes in IFN-α, TGF-β, and GM-CSF levels over time in this study

**Fig. 7:**
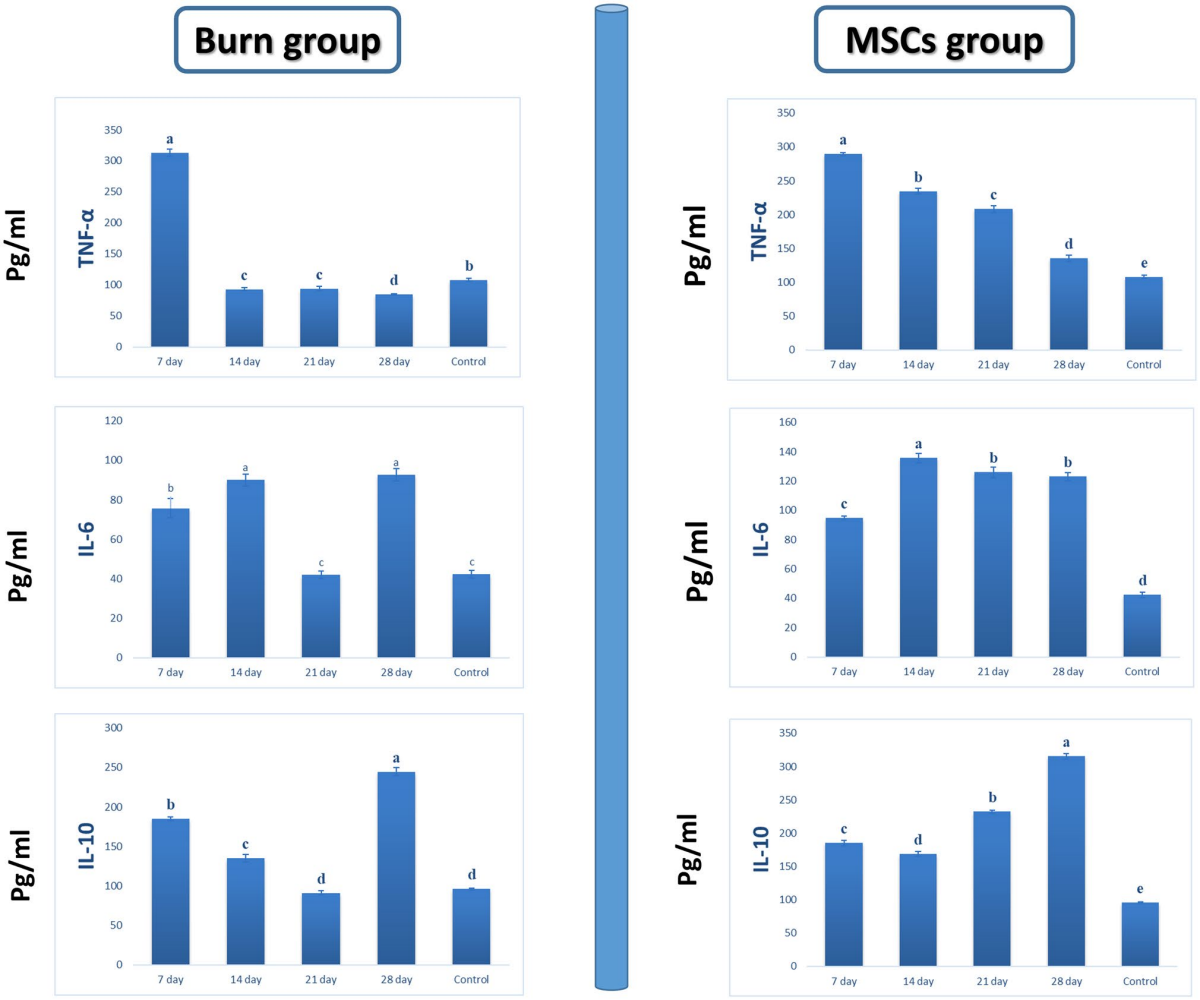
Analysis of the levels of pro-inflammatory and anti-inflammatory cytokines in burned untreated rats, rats treated with MSCs, and control rats through ELISA testing. IL-10, an anti-inflammatory cytokine, inhibits pro-inflammatory cytokines (TNFα and IL-6) in this study. TNF-α and IL-6 are considered pro-inflammatory cytokines here

The two immunomodulatory cytokines (IL-10, TGF-β) in the burned mice showed a great decrease at 7 days post burn, followed by considerable elevations until 28 days, *p* < 0.001 (Figs. 6, 7).

On the other hand, the levels of pro-inflammatory cytokines, such as IL-6 and TNF-α were significantly high in the experimental burn group. In contrast, anti-inflammatory cytokine levels, such as IL-10, in these groups were higher than in the control group (Table 4).

**Table 4:** Comparative Analysis of Interferon-Alpha (IFN-α), Transforming Growth Factor-Beta (TGF-β), Granulocyte-Macrophage Colony-Stimulating Factor (GM-CSF), Tumor Necrosis Factor-Alpha (TNF-α), Interleukin-6 (IL-6), and Interleukin-10 (IL-10) Levels at Various Time Points within the Mesenchymal Stem Cell (MSCs) Experimental Group (n=20), with Subdivision into Four Subgroups of Five Individuals Each per Time Point

Time points	IFN-α	TGF-β	GM-CSF	TNF-α	IL-6	IL-10
7 days (*n* = 5)	22.33 ± 1.53a	561.33 ± 4.16c	15.07 ± 0.9cd	289.33 ± 2.08a	94.87 ± 1.21c	185 ± 4.58c
14 days (*n* = 5)	16.93 ± 0.7b	805 ± 5a	17.5 ± 0.5c	234.67 ± 4.51b	135.67 ± 3.06a	169 ± 3.61d
21 days (*n* = 5)	13.03 ± 0.55d	644.67 ± 5.03b	22.33 ± 2.52b	208 ± 5.29c	126 ± 3.61b	232.33 ± 2.08b
28 days (*n* = 5)	14.53 ± 0.5cd	511.33 ± 1.53d	26.03 ± 1.05a	135.67 ± 4.51d	123 ± 2.65b	315.33 ± 4.51a
*F* ratio	36.84	352.03	48.02	1018.62	612.18	1655.96
*P* value	< 0.001 HS	< 0.001 HS	< 0.001 HS	< 0.001 HS	< 0.001 HS	< 0.001 HS

Significance levels are denoted by letters (a–d) within the same row, where different letters indicate statistically significant differences between the corresponding time point and the control group. Highly significant differences are indicated by HS at *p* < 0.001. Fisher’s test was employed for significance testing at *p* < 0.05

## Discussion

Burn injuries are a major health concern worldwide and a primary cause of illness and death on a global level. Standard treatment for burns typically involves the use of dressings and topical agents that establish a safeguard and facilitate the healing of wounds. Yet, the use of MSCs has emerged as a viable and optimistic substitute for treating burns. This study showed that MSCs have the potency to be used therapeutically to cure rat skin burns. Furthermore, results have shown that local transplantation of MSCs into burn wounds can significantly improve wound healing and skin regeneration, which could have important implications for treating burn injuries in humans.

Initially, the culture of BM-MSCs consisted of a diverse group of cells, while subsequent passages of the culture predominantly exhibited fibroblastic cells. These findings are consistent with previous research conducted on bovine (Corradetti et al. 2013), caprine (Pratheesh et al. 2014), canine (Csaki et al. 2009), and humans (Klemmt et al. 2011).

Nevertheless, our findings regarding the characterization of stem cells in cattle differ from the results reported in other studies (Rossi et al. 2014), where round and fibroblastic cells persisted throughout the culture, even at different stages. This study utilized CD34, CD73^+^, CD90^+^, and CD105^+^ surface antigen markers to assess all three BM-MSCs, consistent with the guidelines established by the ISCT, despite the use of additional marker subsets in other studies (Dominici et al. 2006).

For the best results in wound healing, it is necessary to have a coordinated and effective sequence of three phases that overlap with each other: inflammation, proliferation, and remodeling (Gurtner et al. 2008). Growth factors, cytokines, and chemokines mediate a wide range of cellular and molecular activities. These findings reveal that MSCs significantly enhanced wound healing rates. On the 14th day after the incision, there was a significant improvement (*p* < 0.001) in wound contraction in the groups that received MSC treatment compared to the control group. Similarly, wound contraction at days 21 and 28 was obviously (*p* < 0.001) better in the MSC-treated groups. Also, histopathological examination showed better results for the healed wound in MSC groups (in terms of collagen deposition, neovascularization, epithelization, and collagen assembly) than in the control group. MSCs have been proposed to enhance tissue repair through both paracrine signaling and their ability to differentiate.

While MSC paracrine signaling pathways control local cellular responses to injury, MSC differentiation contributes through the regeneration of damaged tissue (Hocking and Gibran 2010). Endogenous MSCs, such as those seen in skin sheaths and hair follicle bulges, are essential for the healing of wounds (Liu et al. 2014). These cells are distributed across the skin’s many niches, which are primarily classified into epidermal and dermal niches. MSCs play a crucial role in creating a conducive microenvironment for coordinated cellular and molecular activities, such as cell migration. This is achieved through the secretion of factors, macrovesicles, and exosomes by the MSCs (Hocking and Gibran 2010).

Recent research suggests that during the cultivation phase, MSCs release various substances essential for the cell’s regular physiological activity. Additionally, through a procedure called licensing, the innate MSCs are attracted to the wound site and stimulated by the inflammatory environment as they engage with the immune system (Shi et al. 2012) and begin to produce growth factors and cytokines resulting in compositional changes of local cytokines that are conducive to wound healing and tissue regeneration processes (Tamama and Kerpedjieva 2012). In our recent study, we observed a rapid increase in the levels of pro-inflammatory cytokines (TNF and IL-6) in response to short-term inflammation. However, we also found that BM-MSCs significantly counteracted these changes by quickly increasing in number.

Through paracrine mechanisms, wound healing improves by inhibiting the increasing angiogenesis, inflammatory process, collagen production, and stimulating fibroblast migration (Tamama and Kerpedjieva 2012). Furthermore, their paracrine factors suppress nucleic acid, protein metabolism, and apoptotic genes while increasing homeostatic and anti-apoptotic genes (Wu et al. 2006).

After severe burn damage, the systemic inflammatory response triggers the hypermetabolic response, which turns on protein breakdown and catabolism (Jeschke et al. 2008). As a result, pro-inflammatory mediators are released out of control, which worsens organ dysfunction and protein loss (Jeschke et al. 2002; Yeh et al. 2002). The failure of the organ’s performance increases the chances of contracting an infection and sepsis, ultimately failing multiple organs and eventual demise (Agarwal et al. 2023). This vicious circle is not well comprehended and challenging to overcome. There is a lack of testing for treatments that can alter the body’s response to burns, such as inflammation, hypermetabolism, or organ damage, in humans. While we have established a cytokine expression pattern in children who have sustained burns, further research is needed to find effective solutions (Finnerty et al. 2006); in animal research models used for burn pathophysiology, little information is known about the cytokine cascade’s duration or amplitude following an intense thermal injury.

After skin injury, a series of collaborative and dynamic processes occur in a well-coordinated manner to repair and regenerate the skin’s protective ability. These processes involve hemostasis, inflammation, proliferation, and remodeling (Reinke and Sorg 2012). After treatment with BM-MSCs, indicators of wound healing, such as the acceleration of re-epithelialization and thickness of the regenerated epidermis, were noted (Fu et al. 2006). To promote regeneration following burn injury, BM-MSCs are implanted into the damaged area and interact with the epithelial cells while transdifferentiating (Seppanen et al. 2013).

The investigation revealed that BM-MSCs can expedite the healing of skin wounds by promoting the production of collagen bundles, fibroblasts, basal cell proliferation, and vascularization, as well as exhibiting anti-inflammatory properties.

The results obtained by stereological analysis show that using BM-MSCs promotes the formation of the fibrous, thick dermis and granular tissue during epithelialization, increases collagen synthesis, and reduces the inflammatory cell numbers in the wound area.

Moreover, the investigation demonstrated that significant enhancement in vessel length and wound closure area was observed on days 21 and 28 following the introduction of burn injury, as compared to the group treated with BM-MSCs and other groups. The utilization of MSCs boosts the number of basal cells, fibroblasts, blood vessels, and fibrous tissue while reducing the count of inflammatory cells (neutrophils and lymphocytes), thereby facilitating more effective wound healing. Our histological observations are in close alignment with those of the study by Aryan et al. (2018). These findings provide strong evidence that MSCs can promote the proliferation of fibroblasts, which leads to intensified granulation tissue formation, the accumulation of collagen fibers, increased blood vessel formation, and improved re-epithelialization, contributing to enhanced wound healing.

The healing process of wounds consists of three phases—inflammation, proliferation, and remodeling—which occur concurrently and overlap with each other (Cañedo-Dorantes and Cañedo-Ayala 2019).

The extent and comprehension of the inflammatory reaction to burns in mice are limited. Moreover, the expression of cytokines in the blood plasma or serum of uninfected burned rodents is not well established. The typical cytokines measured after a burn injury in mice are IL-1, IL-6, or TNF, and the available literature presents conflicting results regarding the expression of these substances. In one particular study, mice with burns were found to have elevated serum levels of IL-6 and IL-1b after 24 h (Ipaktchi et al. 2006). On the basis of a subsequent inquiry, TNF, IL-6, and IL-10 were not detected in mice that had been burned or those that had undergone a sham procedure, during the first and seventh days following the burn (Murphy et al. 2005).

In contrast, we observed the presence of the three cytokines during days 7 and 14 after the burn was initiated. The number of burns in these two investigations differs significantly from ours; both experiments were conducted on mice that burned more than 25% (Murphy et al. 2005), 30% (Ipaktchi et al. 2006), and 35% total body surface area (TBSA) in this case. Patients’ inflammatory responses vary with burn size (Jeschke et al. 2007). Therefore, these variations in burn size could cause an apparent discrepancy in the data. The patterns we show for rats are supported by other research on rats that burnt more than 20% TBSA. Less than 72 h after burns, elevated levels of IL-1, TNF, and IL-6 have been noted (Kataranovski et al. 1999); our results found the same elevations in rats with burns. Rats burnt with above 60% TBSA showed an increase in IL-1b, IL-6, and IL-10 within hours of burn damage but no change in TNF, according to Gauglitz et al. (2008). Nonetheless, several cytokines that are not commonly evaluated in mice with burns have not been previously documented in terms of their adjustment after burns (Gauglitz et al. 2008).

The present study aimed to achieve two objectives. Firstly, to evaluate the cytokine expression in the serum of rats that were burned and compare it to the cytokine expression in normal, non-burned control rats and rats that underwent local transplantation of MSCs. Secondly, to assess the cytokine profile in burned rats that experienced altered expression after the burn injury, as these cytokines play a crucial role in the proliferation, differentiation, and clonal expansion of immune cells. Furthermore, these cytokines attract immune cells to the injury site. However, the massive upregulation of both pro-inflammatory and anti-inflammatory cytokines may cause non-specific inflammation, rather than a well-planned systemic inflammatory response where these two types of cytokines regulate each other in a coordinated manner (Li et al. 2020).

MSCs could modulate the immune system and reduce inflammation by inhibiting the production of pro-inflammatory cytokines such as TNF and IFN while increasing the secretion of anti-inflammatory cytokines such as IL-10 and IL-4. This property, combined with their inhibitory effect on neutrophil infiltration and IL-6, facilitates successful wound healing through the resolution of inflammation. In studies involving burnt animals, the injection of BM-MSCs significantly reduced the levels of IL-6 and TNF. The current study on a deep second-degree burn also reported an increase in TGFβ expression (Aggarwal and Pittenger 2005; Caliari-Oliveira et al. 2016; Gilbert et al. 2016).

In addition to their inhibitory effect on neutrophil infiltration and IL-6, the immunomodulating property of MSCs enables them to directly reduce the inflammatory response by inhibiting the production of pro-inflammatory cytokines like TNF and IFN and increasing the secretion of anti-inflammatory cytokines like IL-10 and IL-4. Successful wound healing is achieved through the resolution of inflammation. As a result, after subcutaneous injection of BM-MSCs in the burnt animals, the rate of production IL-6 and TNF was considerably reduced. We found an upregulation of TGF-β expression, which was reported in the current study of a deep second-degree burn.

Owing to their capacity to activate dermal fibroblasts, which increase the production of collagen type I and alter gene expression (Smith et al. 2010), MSCs speed up the healing of wounds (Rodrigues et al. 2019). In the current study, it was found that MSCs can hinder scarring by releasing VEGF and HGF, as well as by controlling the equilibrium between TGF1 and TGF3 through their paracrine signaling, as reported by (Colwell et al. 2005). Hsp90 plays a crucial part in this process by boosting cell survival and motility, and growth factors are not the only variables involved in wound closure (Li et al. 2012; Dong et al. 2016). Furthermore, Cheng et al. (2008) and Abdallah et al. (2023) found that it was accountable for the relocation of human epidermal and dermal fibroblasts. As for the process of epithelization, BM-MSCs enhanced this mechanism by promoting the proliferation of resident epidermal cells with the aid of EGF, or through their capacity to transform into epidermal cells (Kataoka et al. 2003; Rodrigues et al. 2019).

Our research reveals the concurrent involvement of multiple cytokines in the rat’s response after burns, marking the first instance of such a phenomenon. Additionally, we provide evidence indicating that six out of 11 cytokines exhibit comparable expression patterns throughout the inflammatory response to burns in rats, also a novel finding. Differences in the post-burn inflammatory response between mice, humans, and rats could be attributed to variations in species as well as the extent and severity of the burn. Moreover, we demonstrate that the duration and magnitude of the inflammatory response to burns in rats are like those documented in humans by Cui et al. (2023).

Previous research has shown that local injection of MSCs along with growth factors can affect the levels of pro-inflammatory and anti-inflammatory cytokines in the serum of rats that have undergone burn injury and local transplantation (Liu et al. 2020; Abdel-Gawad et al. 2021). The present study, on the other hand, focused on the impact of developmental factors injected in MSCs, which led to a significant decrease in the levels of pro-inflammatory cytokines such as TNFα and IL-6, while elevating the levels of anti-inflammatory cytokines, specifically IL-10 and TGF-β, in the serum of rats with burn injuries.

In prior research, skin injuries that received human umbilical cord MSCs transplantation exhibited a notable decrease in the number of inflammatory cells and pro-inflammatory cytokines when compared to the group that received PBS treatment (Liu et al. 2014).

Moreover, the rise in cytokines that have an anti-inflammatory effect, such as IL-10 and TGF-β, might be attributed to the transformation of MSCs into cell types, like fibroblasts, which are crucial in the restoration and regeneration of tissues. Furthermore, the secretion of growth factors, such as VEGF, that stimulate the development of new blood vessels and tissue regeneration, could occur because of the differentiation of MSCs into fibroblasts.

According to our research, one of the significant outcomes was the capacity of MSCs to stimulate the generation of growth factors, such as VEGF and TGFβ, which play a crucial role in tissue repair and wound healing. Another important finding was that the increased production of these growth factors, as observed in the histological analysis of treated wounds, accelerated the healing process and improved tissue regeneration (Oryan et al. 2019).

Additionally, we observed a reduction in inflammation and modulation of the immune response following local injection of MSCs, which is critical in the pathogenesis of burn injuries. This outcome implies that MSCs could potentially have a significant immunomodulatory effect, which could contribute to the improved healing observed in our experiments.

The potential of MSCs for medicinal and regenerative purposes has made them highly sought-after. MSCs can differentiate and repair damaged epithelium through differentiation and fusion, as well as release a diverse range of growth factors and cytokines (Li et al. 2023). Utilizing MSCs-conditioned medium (CM), which contains a wealth of growth factors and cytokines, is advantageous as it eliminates the risk of transplant rejection (Csaki et al. 2009). However, there is currently limited information regarding the comparison of MSC-CM in wound healing (Saadh et al. 2023).

In brief, the findings of this study indicate that providing growth factor-infused MSCs directly to burned rats’ injury sites can shift the balance of pro-inflammatory and anti-inflammatory cytokines in their blood, resulting in improved tissue healing and reduced inflammation. However, further investigation is required to determine the optimal dosage and timing of MSC injection to maximize therapeutic benefits.

To summarize, the study suggests that injecting MSCs with growth factors locally can be a useful therapy for burn wounds, as it reduces inflammation and promotes tissue repair. The decrease in pro-inflammatory cytokines such as TNF-α and IL-6 may be due to the immunomodulatory properties of MSCs, which suppress the activation and proliferation of immune cells like T, B, and natural killer cells. Moreover, MSCs release anti-inflammatory cytokines such as IL-10 and TGF-β, which suppress the production of pro-inflammatory cytokines.

## Conclusion

The results of local injection of MSCs for burn treatment suggest that this approach could significantly influence the field of regenerative medicine. MSCs could become a widely used therapy for burn injuries in humans, providing a more effective and efficient way to promote wound healing and tissue regeneration. Furthermore, the future of regenerative medicine appears bright, with MSCs playing a vital role in developing and progressing new and innovative therapies.

## Data Availability

The datasets generated and analyzed during the current study are available from the corresponding authors on reasonable request.
